# Colorimetric Assay and Antibacterial Activity of Cotton, Silk, and Wool Fabrics Dyed with Peony, Pomegranate, Clove, *Coptis chinenis* and Gallnut Extracts

**DOI:** 10.3390/ma2010010

**Published:** 2009-01-15

**Authors:** Young-Hee Lee, Eun-Kyung Hwang, Han-Do Kim

**Affiliations:** 1 Department of Organic Material Science and Engineering, Pusan National University, Busan 609-735, Korea. E-Mail: youngheelee@pusan.ac.kr; 2 Korea Silk Research Institute, Jinju 660-904, Korea. E-Mail: pukpuck@hanmail.net

**Keywords:** Antibacterial activity, Natural colorants, Colorimetric assay

## Abstract

To investigate the antibacterial functionality of natural colorant extracts, five kinds of natural dying aqueous solutions were obtained by extraction from peony, pomegranate, clove, *Coptis chinensis* and gallnut using water at 90 °C for 90 min with a liquor ratio (solid natural colorant material/water, weight ratio) of 1:10. The colorimetric assay and antibacterial activity of cotton, silk, and wool fabrics dyed with these natural colorant extracts were examined. It was found that these properties were significantly dependent on the structure of colorant and the kind of fabrics. The hues (H) of all fabrics dyed with these natural colorants were in the range of 6.05YR -1.95Y. The order of value (V) was wool, silk and cotton. The chroma (C) of all samples was found to be at very low levels indicating the natural tone. All the fabrics dyed with the five natural colorants (peony, pomegranate, clove, *Coptis chinensis* and gallnut) extracts displayed excellent antibacterial activity (reduction rate: 96.8 - 99.9%) against *Staphylococcus aureus*. However, in the case of *Klebsiella pneumoniae*, the antibacterial activity was found to depend on the kind of natural colorant extract used.

## 1. Introduction 

Recently there has been a revival of interest in the use of natural dyes in textile coloration. This is a result of the stringent environmental standards imposed by many countries in response to the toxic and allergic reactions associated with the use of synthetic dyes. A widespread interest has emerged in the dyeing of textile fibres using natural colorants, on account of their high compatibility with environment, softer colour shades, naturalness, lower toxicity and antibacterial/anti-allergic/deodorizing/anti-cancer properties, harmonising natural shades or just the novelty [1,2,3,4,5,6]. 

It is well known that problems in dyeing with natural dyes are the low exhaustion of natural colorants and the poor fastness of dyed fabrics. Attempts to overcome these problems have been mainly focused on the use of metallic salts as mordants, which are traditionally used to improve fastness properties or exhaustion and to develop different shades with the same dye [7,8,9,10,11,12]. However, most synthetic mordants are toxic heavy metal ions, which are also pollutants. Reports on dyeing properties using natural colorants without mordants are currently not available in the open literature, which makes achievement high exhaustion and good fastness using natural colorants without mordants all the more significant. 

In our previous studies, the dyeability, fastness and deodorizing properties of cotton, silk, and wool fabrics dyed with some natural colorants (sappan wood, black tea, peony, clove, gardenia, coffee sludge, *Cassia tora. L*., and pomegranate) without mordant were investigated [13,14,15]. These properties were found to be closely dependent on the extract concentration, colorant structure and fabric type. The fastness (light, water, and perspiration fastness: 2^nd^ - 5^th^ grades) and the deodorizing performance (34-99%) were found to be related to the K/S and the combination between fabrics and natural colorant extracts. It was found that the use of some natural colorants notably enhanced the deodorizing performance. 

As living standards have improved, people have become extremely concerned about health and hygiene. An increasing volume of literature demonstrates the survival and growth of harmful microorganisms (*Staphylococcus epidermidis, Staphylococcus aureus, Neisseria, Bacillus subtilis, Diphtheroid bacilli, Escherichia coli, Pseudomonas aeruginosa, Alcaligenes faecalis, Klebsiella, Enterobacter aerogenes, Citrobacter and Candida albicans*) in textiles and the health risks of their dissemination [16]. Major antibacterial agents for textiles include metals, metal based compounds, phenolic compounds and quaternary ammonium salts, etc. which all have toxicity and environmental issues. It has become increasingly important for antibacterial agents to meet environmental and low toxicity criteria, while retaining their functionality. Therefore, it is vital to research and develop eco-friendly antibacterial agents extracted from plants/animals for textile applications. The effect of various plants on bacteria has been studied by a number of researchers [17,18,19]. However, despite the fact that there are many natural antibacterial agents, few studies in the open literature have explored their antibacterial activity on textile materials.

This study investigated the antibacterial functionality of cotton, silk, and wool fabrics dyed using five kinds of natural aqueous dyeing solutions obtained by extraction from peony, pomegranate, clove, *Coptis chinensis*, and gallnut. *Staphylococcus aureus* and *Klebsiella pneumoniae*, the micro-organisms typically known to grow on textiles, were used in this study. The colorimetric assay and antibacterial activity of cotton, silk, and wool fabrics dyed with above natural colorant extracts without mordants were also examined in this work. 

## 2. Results and Discussion 

### 2.1. Confirmation of components of various natural colorant extracts

**Figure 1:**
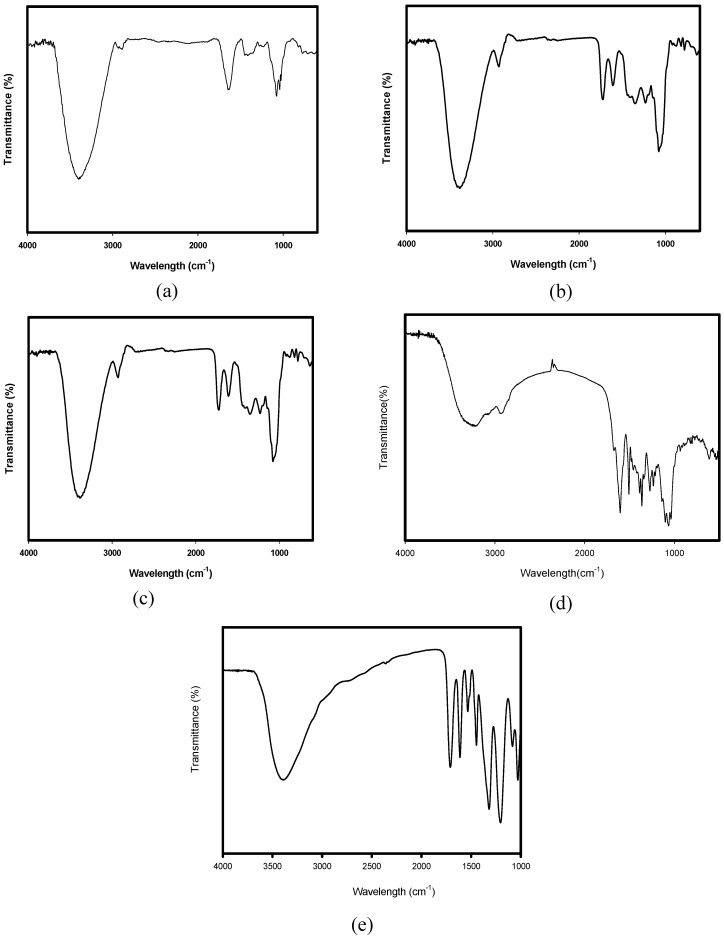
Infrared spectra of extracted natural colorants: peony (a), pomegranate (b), clove (c), *Coptis chinensis* (d) and gallnut (e).

Figure 1 shows the IR spectra obtained for the peony, pomegranate, clove, *Coptis chinensis* and gallnut extracts. 

Peony extract (a) showed characteristic peaks corresponding to the phenol at 3,200 cm^-1^ and 1.238 cm^-1^, phenyl CH stretch at 2,903 cm^-1^, ring stretch (benzene ring in aromatic compounds) at 1,636 cm^-1^ and the C=O stretching at 1,419, 1,086, and 1,040 cm^-1^ were observed, potentially indicating the presence of paenol/paenoside/paeonolide/paenoniflorin in the peony extract.

Pomegranate extract (b) had characteristic peaks corresponding to the OH at 3,384 cm^-1^, CH anti-symmetric and symmetric stretch at 2,926 cm^-1^, C=O stretch at 1,732 cm^-1^, C=C stretch at 1,603 cm^-1^, C-O stretch at 1,071 cm^-1^ were observed. This indicated the presence of ellagic acid in this extract.

Clove extract (c) has the characteristic peaks corresponding to the vinyl =CH stretching at 2,931 cm^-1^, the overtone of vinyl CH_2_ out-of-plane wagging and C=O stretch at 1,724 cm^-1^, ring stretch (benzene ring in aromatic compounds) at 1,608 cm^-1^, CH_3_ antisymmetric deformation at 1,447 cm^-1^ and 1359 cm^-1^ , phenol at 1,220 cm^-1^, C-O stretch at 1,044 cm^-1^ indicating the presence of eugenol in clove extract.

*Coptis chinensis* extract (d) has the characteristic peaks corresponding to the OH stretch at 3,300 cm^-1^, NH deformation at 1,506 cm^-1^ , CH_3_ deformation at 1,387 cm^-1^, -CH_3_ sym deformation at 1,362 cm^-1^, C-O-C antisym stretch at 1,233 cm^-1^, and C-O stretch at 1,075 cm^-1.^ This indicated the presence of berberine, coptisine, worenine in *Coptis chinensis*.

Gallnut extract (e) displayed characteristic peaks corresponding to the OH stretch at 3,382 cm^-1^, C=O stretch at 1,709 cm^-1^, ring stretch at 1,612 cm^-1^, C-O-C antisym stretch at 1,362 cm^-1^, C-O stretch at 1,030 cm^-1^. This indicated the presence of penta-*m*-digalloyl-β-glucose, gallic acid in gallnut. 

Natural colorant extracts are composed of main component and many minor components yet to be characterized fully. The FTIR characterization presented herein can potentially identify components present in the extracts for future studies to determine the source of the extract antibacterial activity. A detailed analysis of each major and minor component will require further characterization by confirmatory chromatographic and mass spectrometric techniques. 

### 2.2. Concentrations of extracted natural colorants

From the results of our previous study, the optimum liquor ratio was found to be 1:10 [15]. Therefore, all natural colorants were extracted at 1:10 liquor ratio in this study. The concentrations of peony, pomegranate, clove, *Coptis chinensis*, and gallnut extracts were found to be 1.71, 0.96, 0.92, 1.92, and 6.41g/100 mL, respectively. The concentration of extract increased in the order of clove < pomegranate < peony < *Coptis chinensis* < gallnut. 

### 2.3. Colorimetric assay

In this study, the dyeing time and temperature were fixed at 60 min and 80 °C, respectively. It was because the preliminary experiment found that the optimum dyeing time and temperature at a fixed liquor ratio (1: 10) were 60 min and 80 °C respectively [15].

**Table 1:** Colorimetric data of fabrics dyed with peony, pomegranate, clove, *Coptis chinensis* and gallnut extracts.

Fabric	Natural colorant	*K/S*	L*	a*	b*	*H*	*V/C*
**Cotton**	**Control**	0.08	88.97	-0.25	1.10		
	**Peony**	0.54	84.47	0.94	5.92	9.48YR	8.34/0.91
	**Pomegranate**	1.18	86.51	0.09	11.22	1.84Y	8.55/1.53
	**Clove**	1.70	80.08	2.40	16.92	0.31Y	7.88/2.61
	***Coptis chinensis***	2.08	76.15	1.81	24.96	1.94Y	7.47/3.73
	**Gallnut**	0.52	88.48	-0.03	8.15	1.95Y	8.75/1.09
**Silk**	**Control**	0.11	86.87	-0.21	1.10		
	**Peony**	1.76	75.13	3.55	7.92	6.05YR	7.36/1.56
	**Pomegranate**	3.18	80.39	1.39	17.13	1.22Y	7.91/2.54
	**Clove**	7.00	64.28	7.38	28.66	9.54YR	6.25/4.78
	***Coptis chinensis***	7.32	64.79	9.39	51.44	0.63Y	6.30/8.11
	**Gallnut**	1.10	84.74	1.5	7.54	8.87YR	8.36/1.20
**Wool**	**Control**	0.27	86.38	-1.23	5.00	-	-
	**Peony**	4.26	66.77	4.40	15.91	8.97YR	6.50/2.67
	**Pomegranate**	5.97	73.97	3.43	33.09	1.72Y	7.24/5.04
	**Clove**	10.46	54.40	11.16	35.03	8.80YR	5.26/5.92
	***Coptis chinensis***	11.01	56.33	10.50	48.57	0.30Y	5.45/7.71
	**Gallnut**	3.34	72.75	3.38	17.67	9.98YR	7.11/2.81

**Figure 2:**
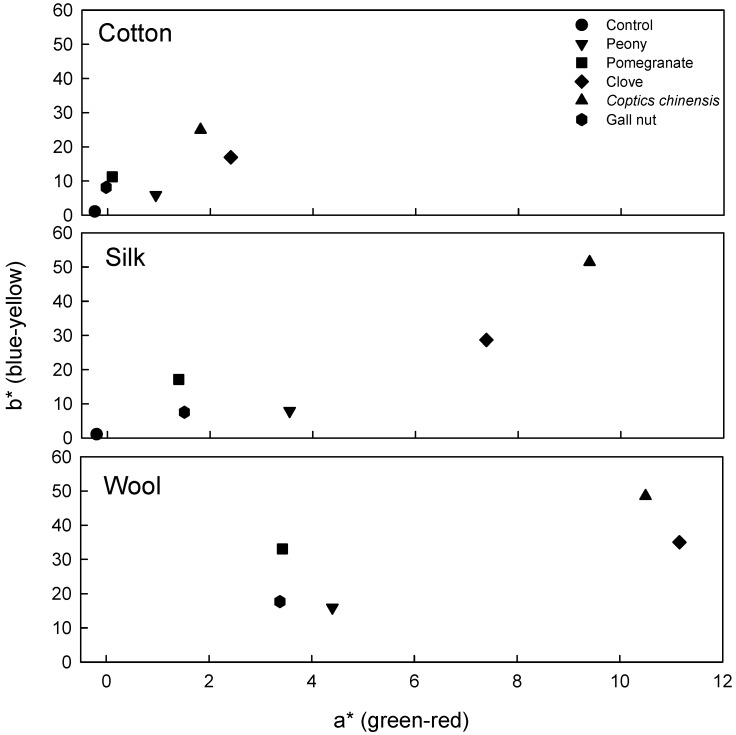
Various colors of dyed fabrics on coordinate a*(greenness-redness) and b*(blueness-yellowness).

Table 1 and Figure 2 show colorimetric parameter (L*, a*, b*, *H and V/C*) of fabrics (cotton, silk, and wool) dyed with peony, pomegranate, clove, *Coptis chinensis* and gallnut extracts. The values quoted are the averages of five measurements. Generally, the dyeing affinity of textile materials is dependent on the content and type/polarity of functional groups of fibers. It is well known that the number of functional group in wool is larger than that of silk, and polarity of protein fibers is higher than that of cellulose fibers [20].

**Figure 3:**
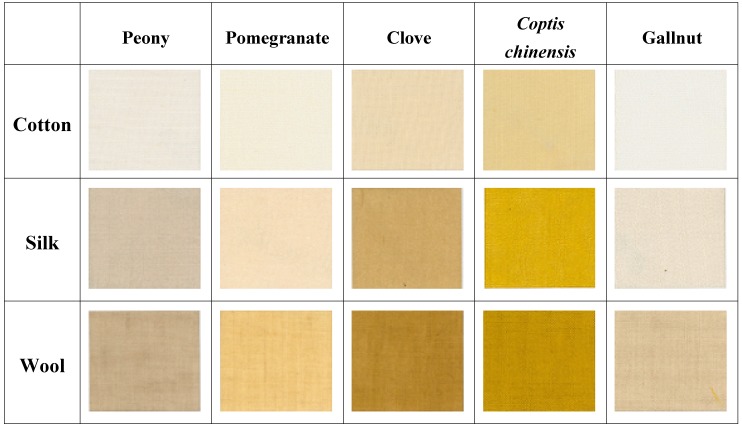
Various colors of dyed fabrics (cotton, silk, and wool fabrics) using peony, pomegranate, clove, *Coptis chinensis* and Gallnut.

The color hues of cotton dyed with peony, pomegranate, clove, *Coptis chinensis* and gallnut were found to be 9.48YR, 1.84Y, 0.31Y, 1.94Y, and 1.95Y, respectively. The color hues of silk dyed with these natural colorants were 6.05YR, 1.22Y, 9.54YR, 0.63Y, and 8.87YR, respectively. The values of wool were 8.97YR, 1.72Y, 8.80YR, 0.30Y, and 9.98YR, respectively. In this study, various color characters of dyed fabrics were characterized using coordinate a*(redness) and b*(yellowness) as shown in Figure 2. Wool showed the highest a* and b* values for all colorants (peony, pomegranate, clove, *Coptis chinensis* and gallnut), followed by silk and cotton, indicating that the increase of redness and yellowness was in the order of wool > silk > cotton (see Table 1 and Figure 2). Various apparent colors of fabrics (cotton silk and wool fabrics) dyed with five kinds of natural colorants are shown in Figure 3. It was found that the color of dyed fabrics was dependent on the kinds of natural colorants and fibers. Various elegant colors were obtained using natural colorant extracts such as peony, pomegranate, clove, *Coptis chinensis* and gallnut extracts (see Figure 3).

### 2.4. Antibacterial activity

**Figure 4:**
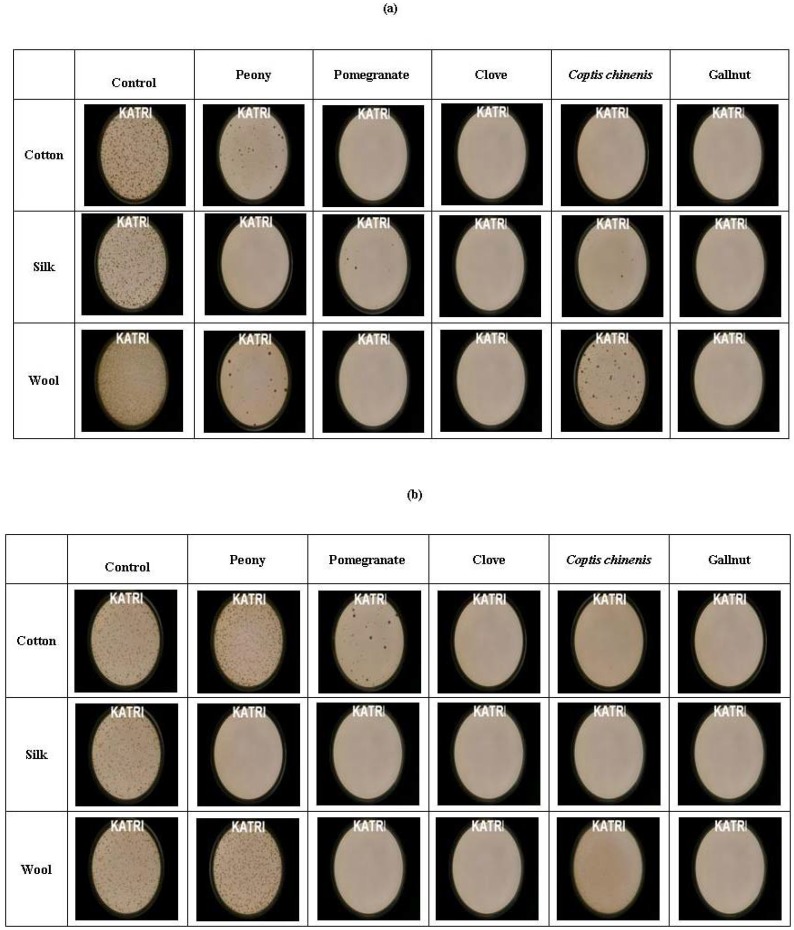
The micrographs of antibacterial activity of control fabrics and dyed fabrics using various natural colorants against (a) *Staphylococcus aureus* and (b) *Klebsiella pneumoniae.*

Figure 4 shows the micrographs of antibacterial activity of control fabrics and dyed fabrics (cotton, silk, and wool fabrics dyed with peony, pomegranate, clove, *Coptic chinensis* and gallnut extracts) against *Staphylococcus aureus* and *Klebsiella pneumonia*. The bacteriostatic reduction rates of control fabric and dyed fabrics against *Staphylococcus aureus* and *Klebsiella pneumonia* are given in Table 2. All the fabrics dyed with the four kinds of natural colorants (pomegranate, clove, *Coptis chinensis* and gallnut) extracts displayed outstanding antibacterial activities against *Staphylococcu aureus* (reduction rate: 96.8 - 99.9%) and *Klebsiella pneumonia* (95.7- 99.9%). All the fabrics dyed with peony extract showed excellent antibacterial activity (reduction rate: 98.8 - 99.7%) against *Staphylococcu aureus*, however, peony did not display antibacterial activity against *Klebsiella pneumonia*. 

The antibacterial activity of pomegranate extract was might be attributable to ellagic acid and tannin components. The antibacterial activity of *Coptic chinensis* extract might be due to the berberine which is known to possess good antibacterial activity. In the case of peony extract, although there was no special component which is known to show good antibacterial activity, the antibacterial activity of peony extract might be due to unknown minor components such as tannins and steroids. In the case of clove, the antibacterial activity might be creditable mainly to the phenol component in eugenol. Natural colorant extracts are composed of main component and many unknown components. Therefore, it is very difficult to identify the exact components that have antibacterial activity against microorganisms. More detailed studies should be made. 

**Table 2:** The bacteriostatic reduction rates (%) of cotton, silk, and wool fabrics dyed with peony, pomegranate, clove, *Coptis chinensis* and gallnut extracts against *Staphylococcus aureus* and *Klebsiella pneumoniae*.

	Cotton		Silk		Wool	
**Natural colorant**	***Staphyl-ococcus aureus***	***Klebsiella pneumoniae***	***Staphyl-ococcus aureus***	***Klebsiella pneumoniae***	***Staphyl-ococcus aureus***	***Klebsiella pneumoniae***
**Pomegranate**	99.9	95.8	99.8	99.5	99.9	99.8
**Clove**	99.9	99.9	99.9	99.9	99.9	99.9
**Peony**	98.8	0.0	99.7	0.0	99.4	0.0
***Coptis*** ***chinensis***	99.9	99.9	99.9	99.9	96.8	95.7
**Gallnut**	99.9	99.9	99.9	99.9	99.9	99.9

## 3. Experimental Section 

### 3.1. Materials

Cotton, silk and wool fabrics (Standard Adjacent Fabrics for Staining of Fastness Test: KS K 0905) were used. The characteristics of these fabrics are shown in Table 3. Peony, pomegranate, clove, *Coptis chinensis* and gallnut extracts were used as natural colorants.

**Table 3:** Characteristics of fabric samples.

		Cotton	Silk	Wool
**Thickness^a^ (mm)**		0.27	0.10	0.33
**Counts (Tex)**	**Warp**	14	2.3/2	15.6/2
	**Weft**	16.5	2.3	15.6/2
**Density (threads/cm)**	**Warp**	31	38	18
	**Weft**	35	55	21
**Weight (g/m^2^)**		115	26	125
***K/S***		0.08	0.11	0.27
L*		88.97	86.87	86.38
a*		-0.25	-0.21	-1.23
b*		1.10	1.03	5.00
**WI**		68.51	64.34	43.65
**YI**		1.97	1.93	8.69
**BI**		73.08	68.88	64.76

Weave: plain ^a^Thickness were measured under 1 kpa pressure WI: Whiteness Index, 10deg./D65/Ganz YI: Yellowness Index, 2deg./C/ASTM D1925 BI: Brightness Index, 2deg./C/TAPP1452/ISO2470

### 3.2. Extraction / Dyeing

*Extraction*: Five kinds of dying solutions were extracted from peony, pomegranate, clove, *Coptis chinensis* and gallnut using water at 90 °C for 90 min. at a fixed liquor ratio (solid natural colorant/water ratio) of 1:10.

*Dyeing*: All dyeing was carried out using a 1:100 bath ratio at 80 °C for 60 min by exhaustion method.

### 3.3. Characterization

To confirm the structure of natural colorants, FTIR spectrometer (Impact 400D, Nicolet , Madison, WI) was used to measure the infrared spectra of extract solution in the wavenumber of 400-4000 cm^-1^ at room temperature. For each IR spectrometer samples 32 scans at 4 cm^-1^ resolution was collected in the transmittance mode. 

The reflectance values and the corresponding CIE L*, a*, b*, H V/C, and color strength (*K/S*) values for the dyed samples were measured using a CCM (Gretag Macbath Color-Eye 7000A, USA) interfaced to a digital PC under illuminant D_65_, with a 10^o^ standard observer. K/S was calculated from the reflectance values using the Kubelka-Munk equation as follows:

*K/S = (1-R) ^2^/2R - (1-R_0_)^2^ /2R_0_*

where *R* is the reflectance of the colored fabric, *R_0_* is the reflectance of the uncolored fabric, and K/S is the ratio of the absorption coefficient (*K*) to scattering coefficient (*S*): the higher the value, the greater the colour strength.

The antibacterial activity of the cotton, silk, and wool fabrics dyed with peony, pomegranate, clove, *Coptis chinensis* and gallnut against *Staphylococcus aureus* ATCC 6538 (gram positive) and *Klebsiella pneumoniae* ATCC 4352 (gram-negative) according to modified KSK 0693-2006 (Assessment of antibacterial activity). The concentrations of the cultures were adjusted using spectrophotometer(λ660 nm) to 1.3 x 10^5^ colony forming unit (CFU) per mL. The bacteriostatic reduction rate was estimated by the standard equation: Reduction (%) = [(A-B) / A] x 100, where, A and B is the bacteria colonies of untreated and treated fabrics, respectively.

## 4. Conclusions 

Five kinds of natural dying solutions were obtained by extraction from peony, pomegranate, clove, *Coptis chinensis* and gallnut using water at 90 °C for 90 min with a liquor ratio of 1:10. The colorimetric assay and antibacterial activity of cotton, silk, and wool fabrics dyed with these natural colorant extracts were examined. It was found that these properties were closely dependent on the structure of colorant and the kind of fabrics. The hues of all fabrics dyed with these natural colorants were in the range of 6.05YR – 1.95Y (yellow red – yellow color). The order of V value was wool, silk and cotton indicating that the content of natural colorant in fiber increased in the order of wool > silk > cotton. The chroma (C) of all fabrics dyed natural colorant extracts obtained in this study was found to be very low values indicating their natural tones. All the fabrics dyed with the five kinds of natural colorants (peony, pomegranate, clove, *Coptis chinensis* and gallnut extracts) displayed excellent antibacterial activity (reduction rate: 96.8 - 99.9%) against *Staphylococcus aureus*. All the fabrics dyed with pomegranate, clove, *Coptis chinensis* and gallnut extracts showed outstanding antibacterial activity (reduction rate: 95.7 - 99.9%) against *Klebsiella pneumoniae*, however, peony did not display antibacterial activity. These results clearly demonstrate that utilizing extracted natural colorants as dyeing materials significantly facilitate obtaining quality antibacterial fabrics having various natural colors. 
